# Sustained Effects of a Scaled-Up mHealth and School-Based Intervention for Salt Reduction (EduSaltS) in Schoolchildren and Their Families: 1-Year Follow-Up of a Cluster Randomized Controlled Trial

**DOI:** 10.3390/nu17111845

**Published:** 2025-05-28

**Authors:** Naibo Wang, Puhong Zhang, Yinghua Li, Chen Wang, Feng J. He, Li Li, Yuan Li, Rong Luo, Yuanan Lu, Dezhi Wan, Tian Lu, Lewei Xu, Chaochao Zhu, Lei Wu

**Affiliations:** 1 School of Public Health, Jiangxi Provincial Key Laboratory of Disease Prevention and Public Health, Jiangxi Medical College, Nanchang University, Nanchang 330006, China; 2 Jiangxi Provincial Center for Patriotic Health and Health Promotion, Nanchang 330003, China; 3 The George Institute for Global Health, Beijing 100060, China; 4 Beijing Physical Examination Center, Xicheng District, Beijing 100050, China; 5 Chinese Center for Health Education, Beijing 100011, China; 6 Wolfson Institute of Population Health, Barts and The London School of Medicine and Dentistry, Queen Mary University of London, London EC1M 6BQ, UK; 7 Environmental Health Laboratory, Department of Public Health Sciences, Thompson School of Social Work and Public Health, University of Hawaii at Manoa, Honolulu, HI 96822, USA; 8 Jiangxi Association for Health Education and Tobacco Control, Nanchang 330003, China

**Keywords:** school-based health education, mobile health, reducing salt intake, urinary sodium excretion, blood pressure, sustained effect

## Abstract

**Background**: While the mHealth and school-based scale-up intervention for salt reduction (EduSaltS) effectively reduced salt intake and blood pressure among adults living with participating schoolchildren, the sustainability of these effects remains uncertain. This study aimed to evaluate whether these effects persisted one year post intervention. **Methods**: A one-year follow-up of a cluster randomized controlled trial was conducted, involving 524 children and their 524 adult family members from 20 primary schools. At 24 months, 509 children (97.1%) and 486 adults (92.7%) completed the assessment. Mixed linear models were used to analyze the difference in changes in salt intake between the intervention and control groups at 24 months, compared to baseline and 12 months, as measured by consecutive 24 h urinary sodium excretions. Secondary outcomes included the differences in changes in blood pressure and salt-related knowledge, attitudes, and practices (KAP) scores. **Results**: The adjusted mean difference in changes in salt intake between groups was −0.34 g/24 h (95% CI: −0.94 to 0.26, *p* = 0.265) for children and −0.72 g/24 h (95% CI: −1.48 to 0.05, *p* = 0.065) for adults at 24 months versus baseline. The corresponding differences from 12 to 24 months were −0.09 g/24 h (95% CI: −0.69 to 0.51, *p* = 0.775) for children and 0.29 g/24 h (95% CI: −0.50 to 1.08, *p =* 0.468) for adults. The adjusted difference in changes in adult blood pressure showed a slight, nonsignificant rebound at 24 months. The intervention group maintained significantly higher KAP scores than the control group at both 12 and 24 months. **Conclusions**: The effects of EduSaltS on reducing salt intake and blood pressure in adults diminished slightly one year after the intervention ended. However, sustained improvements in salt-related KAP were observed in both children and adults. Ongoing support is vital to sustain long-term salt-reduction behaviors.

## 1. Introduction

Cardiovascular disease (CVD) remains the leading cause of death and disability worldwide, with hypertension as one of its primary risk factors [1,2,3,4]. Extensive evidence indicates that excessive salt intake not only raises blood pressure but is also strongly linked to the development and progression of various non-communicable diseases (NCDs) [5,6,7]. Globally, this unhealthy dietary behavior contributes to more than 1.8 million deaths annually [5]. In response, the World Health Organization (WHO) has identified population-level salt reduction as one of the most efficient and cost-effective public health strategies. Despite global efforts to promote salt reduction, progress has been limited—largely due to individual taste preferences, entrenched cooking habits, and the widespread consumption of highly and ultra-processed foods, which remain major barriers [8,9]. Digital health technologies, particularly mobile health (mHealth), have shown great promise in supporting behavioral changes [10,11], and in expanding both the reach and impact of health education initiatives. Accordingly, comprehensive salt-reduction strategies that incorporate innovative digital health clinicals hold significant potential for public health advancement.

Schools serve as a vital platform for health education and have proven effective in addressing various public health challenges, including smoking, obesity, and CVD [12,13,14]. Leveraging this setting, our research team adopted the “small hands leading big hands” approach—engaging children to actively influence and motivate their parents toward healthier behaviors. Building on this strategy, we conducted a series of studies. One such initiative, the School-EduSalt cluster randomized controlled trial (cRCT), demonstrated significant reduction in salt intake among both children and their parents. However, its reliance on specialized offline activities and intensive teacher training posed challenges for large-scale implementation [15]. To improve feasibility and scalability, we developed AppSalt, a mobile health (mHealth) platform that delivers standardized online health education courses paired with interactive homework assignments aimed at promoting salt reduction within families. A subsequent cRCT evaluating AppSalt confirmed its effectiveness in significantly reducing salt intake and lowering blood pressure among adults [16]. Building on these findings and with the goal of developing a more scalable intervention suitable for integration into routine school health education, an updated program—EduSaltS—was designed and implemented for the first time across three provinces in China: Ganzhou (Jiangxi Province), Zhenjiang (Jiangsu Province), and Qinhuangdao (Hebei Province). EduSaltS incorporates a WeChat-based interactive system, engaging cartoon-enhanced educational content, customized user interfaces for different participant roles, and a comprehensive blend of online and offline health education methods to facilitate broader real-world implementation. The evolution from School-EduSalt to AppSalt and ultimately to EduSaltS is depicted in Figure S1. A process evaluation using the RE-AIM framework demonstrated that EduSaltS achieved broad reach, high acceptability, effective implementation, and promising potential for nationwide scale-up [17].

Although EduSaltS intervention has been validated in real-world settings—demonstrating a reduction in salt intake by 1.06 g/day and preventing a 2.26 mmHg increase in systolic blood pressure among adults [18]—its long-term sustainability remains uncertain. Previous studies have indicated significant challenges in maintaining healthy behaviors over time [19], and evidence from salt-reduction interventions specifically has revealed rebounds in salt intake and blood pressure levels once the intervention ends [20,21]. Therefore, this study aims to evaluate the sustained effectiveness of EduSaltS in schoolchildren and their families one year after the original cluster randomized controlled trial was completed.

## 2. Materials and Methods

### 2.1. Study Design and Participants

The overall design and effectiveness evaluation of the EduSaltS intervention have been published elsewhere [18,22]. Building on the original EduSaltS cluster randomized controlled trial (cRCT), which recruited participants in April 2022 and completed baseline assessments in June 2022, this study assessed the sustainability of intervention effects one year after the cRCT concluded in July 2023, with follow-up conducted in July 2024. The study was conducted in 20 public primary schools across four districts/counties (Zhanggong District, Nankang District, Xinfeng County, and Yudu County) in Ganzhou City, Jiangxi Province, China, with schools randomly assigned to either intervention or control group (1:1) after the baseline survey by the research team using a computer-generated central randomization system, stratified by school location (14 schools in urban districts and 6 in counties classified as rural). The final school assignments are detailed in Table S1. Trained data collectors from hospitals and the Centers for Disease Control and Prevention (CDCs) were blinded to group allocation.

One class of third-grade schoolchildren was randomly selected from each of the 20 participating schools (10 intervention and 10 control). Within each selected class, 26 students were randomly chosen to participate in the study. Invitation letters and study information sheets were distributed to parents through class teachers. If the child’s parent was unavailable, another adult household member (e.g., grandparent) who lived with the child and shared meals was invited to participate in their place. The inclusion criteria for children and their accompanying adult were (1) being willing to participate in the follow-up; (2) no intention for school transfer during the intervention period for children; (3) adult participants aged between 18 and 75 years; and (4) being able to provide regular feedback and complete required outcome assessments. Exclusion criteria included (1) medical conditions or other factors preventing children from participating in assessments or intervention activities; (2) inability or explicit unwillingness to complete follow-up; and (3) inability or unwillingness to collect 24 h urine samples.

### 2.2. Procedure

Following the selection of schools and classes, class teachers distributed introductory materials and consent forms to students and their parents or grandparents. Baseline data collection included the following components: (1) sociodemographic characteristics (e.g., age, gender, education level, and relationship to the participating children) and behaviors (e.g., smoking, alcohol consumption, and physical exercise); (2) measurements including height, weight, and blood pressure, which were conducted using standardized tools by trained CDCs and township health centers staff; (3) salt-related knowledge, attitudes, and practices (KAP) surveys designed specifically for children and adults; and (4) a single 24 h urine collection conducted on a weekday for both children and adults simultaneously. Each participant received four 1000 mL containers and was instructed to collect all urine over a 20–28 h period. The total urine volume was then standardized to reflect a 24 h collection. Detailed instructions were provided both orally and in printed form to ensure clarity. During the 24 h urine collection period, class teachers also sent reminders through the online group to ensure compliance. After 24 h of urine collection, participants were instructed to bring the complete urine sample to the investigator at the designated field site. The start and end times of the collection were carefully recorded. After thorough mixing, the volume of each sample was measured, and the samples were extracted into 2 × 2 mL aliquots, frozen at −20 °C, and then transported to a central laboratory (KingMed Diagnostics, Guangzhou, China) for analysis.

After completing the baseline assessment in June 2022 (at the end of grade three for schoolchildren) and randomization into the intervention and control groups, the EduSaltS intervention was conducted throughout one year. The quality control of the intervention was primarily guided by the research team, with implementation carried out by teachers who received standardized training. The overall intervention was conducted with the joint participation of students, their parents, and schools.

Follow-up assessments, employing the same procedures and methods as at baseline, were performed after the intervention (at 12 months) and again one year later (at 24 months). All individuals who participated at baseline were re-contacted for the 24-month follow-up, regardless of whether they had participated in the 12-month assessment.

### 2.3. Intervention

The EduSaltS intervention lasted for one year, combining online courses, offline interactive activities, and the establishment of supportive low-sodium environments. The online component featured salt-reduction educational content delivered through a WeChat Mini Program (“Health Cloud Classroom”). Each week, students and family members watched an approximately 10-min online health education video, listened to salt-reduction broadcasts, and completed 5–10 interactive quizzes and practical salt-reduction activities. Additional optional modules encompassed nutritional education on diverse food types, interactive quizzes on related knowledge for entertainment, and self-reported dietary records for sodium intake assessment. The offline component primarily involved interactive health education sessions and themed class meetings or activities integrated with routine health education curricula. Each semester, schools were required to organize at least four lectures or themed events on salt reduction. Additionally, EduSaltS facilitated a supportive low-sodium school environment through initiatives such as displaying salt-reduction posters and banners on campus, playing salt and health broadcast series and radios in school, and providing cafeteria staff and catering providers with educational materials aimed at reducing sodium content in school meals.

Students in the control group received routine health education classes, which did not include specific content on salt reduction or involve family engagement. A detailed description on EduSaltS intervention, including its application and web interface, is available in our previous publications [18,22].

### 2.4. Outcomes

The primary outcome of this study was the difference between the two groups in the change of salt intake among children and adults at 24 months versus baseline and 12 months, as assessed through 24 h urinary sodium excretion, which is the most accurate method to estimate salt intake. The secondary outcome was the difference in the change of blood pressure and the change of salt-related knowledge, attitude, and practice (KAP) at 24 months versus baseline and 12 months. All measurements and data collections were conducted once at baseline, after completion of the intervention (at 12 months), and 12 months after the end of intervention (at 24 months). Outcome data for both groups were collected, using the same protocol as the original trial, by trained researchers from the CDCs and hospitals through an electronic data collection system.

#### 2.4.1. Assessment of Salt Intake and Urinary Excretions

Salt intake was assessed via 24 h urinary sodium excretion from consecutive 24 h urine collections, which reflected total dietary sodium intake. This method is considered the gold standard for assessing salt intake. Following the measurement of total urine volume, the sample, after being thoroughly mixed, was analyzed for sodium concentration. This value was then multiplied by the total urine volume (adjusted for any deviations from the 24 h collection period) to determine the 24 h urinary sodium excretion. Salt intake was estimated by multiplying the 24 h urinary sodium excretion by 23 (the atomic weight of sodium) and 2.54 (the sodium-to-salt conversion factor). All samples were tested consistently by a central laboratory (KingMed Diagnostics), with the testing parameters encompassing urinary sodium, potassium, and creatinine concentrations.

#### 2.4.2. Assessment of Blood Pressure and Salt-Related KAP

Blood pressure was measured on the right arm using calibrated Omron electronic sphygmomanometers after a 10-min seated rest. Three measurements were taken with at least a 1-min interval, and the average of the last two readings was used for analysis. Body weight and height were measured using standardized tools. For KAP scores, we updated the KAP questionnaire that was used in previous study and was validated through the pilot study [16]. This questionnaire comprised two to six questions in each dimension (knowledge, attitude, and practice) tailored separately for schoolchildren and adults, with each question scored out of 10 points. After summing scores within each dimension, the average score was calculated by dividing by the number of questions, resulting in a score ranging from 0 to 10 per dimension, with a total possible score of 30 across all three dimensions. Detailed questionnaire items and scoring criteria for schoolchildren and adults can be found in Tables S2 and S3. A higher KAP score indicates greater awareness and improved practices related to salt.

### 2.5. Statistical Analysis

A sample size of 520 students and 520 parents (26 participants per cluster across 10 intervention and 10 control schools) was estimated to provide adequate statistical power to detect a target mean reduction of 1 g/day in salt intake. This calculation assumed a standard deviation of 2.15, an intraclass correlation coefficient (ICC) of 0.01 [15], a significance level of 0.05 and power (1-β) of 0.9, accounting for an anticipated 10% loss to follow-up.

The sustained effect of EduSaltS was assessed using an intention-to-treat analysis without imputation given the low dropout rates at 24 months (2.9%, 15/524 in children; 7.3%, 38/524 in adults). Considering the hierarchical structure of the data, with three measurements (baseline, 12 months, and 24 months) per participant and participants clustered within schools, mixed linear models with random intercepts were employed to analyze the effects of the intervention. Fixed-effect variables incorporated time, group, time × group interaction, and covariates. Potential confounding variables adjusted as covariates included district/county areas, age, gender, BMI (body weight in children instead), education level (for children, it was represented by their familial participants), and physical activity (categorized as “yes” if someone engaged in moderate physical activities at least three times per week for over 30 min each time). Additionally, outdoor temperature was also included as a confounding factor when analyzing blood pressure outcomes. For adult participants, further adjustments were counted for smoking status, alcohol consumption, and familial relationship with children. The differences between the two groups at 24 months compared to baseline and 12 months were analyzed through designated LSMESTIMATE command within these models.

According to previous studies, urine samples were considered likely incomplete and excluded from the primary analysis if (1) 24 h urine volume was <300 mL in children; (2) 24 h urinary creatinine was lower than the 5th percentile (<2.43 mmol for girls and <2.52 mmol for boys); (3) 24 h urine volume was <500 mL for adults; or (4) 24 h urinary creatinine was <4.0 mmol for women or <6.0 mmol for men. The urine sample would also be excluded if the collection time was <20 h or >28 h [15,16]. For the included urine samples, the standardized 24 h urine volume (mL) was determined by dividing the recorded urine volume (mL) by the total collection duration (hours) and then multiplying by 24. The 24 h excretion of sodium, potassium, and creatinine was calculated by multiplying their respective urinary concentrations by 24 h urine volume.

Subgroup and sensitivity analyses were conducted in this study. Subgroup analyses of salt intake and blood pressure were performed to assess the differential effects across various population segments. For both students and adults, differences were evaluated by gender and area (district/county level). For adults, additional subgroup analyses were performed by age groups, education level (primary or below, secondary education, high school, college, or above), blood pressure status (hypertension defined as systolic blood pressure (SBP) ≥ 140 mmHg or diastolic blood pressure (DBP) ≥ 90 mmHg or self-reported hypertension), smoking, BMI (<24.0 kg/m^2^ or ≥24.0 kg/m^2^), and familial relationship with participating children (parent, grandparent, or other). For the sensitivity analysis, potentially incomplete 24 h urine samples were included using an intention-to-treat approach to assess the robustness of the primary findings.

Statistical analyses were performed using SAS 9.4. All tests were two-sided, and a *p*-value of <0.05 was considered statistically significant.

## 3. Results

**Figure 1:**
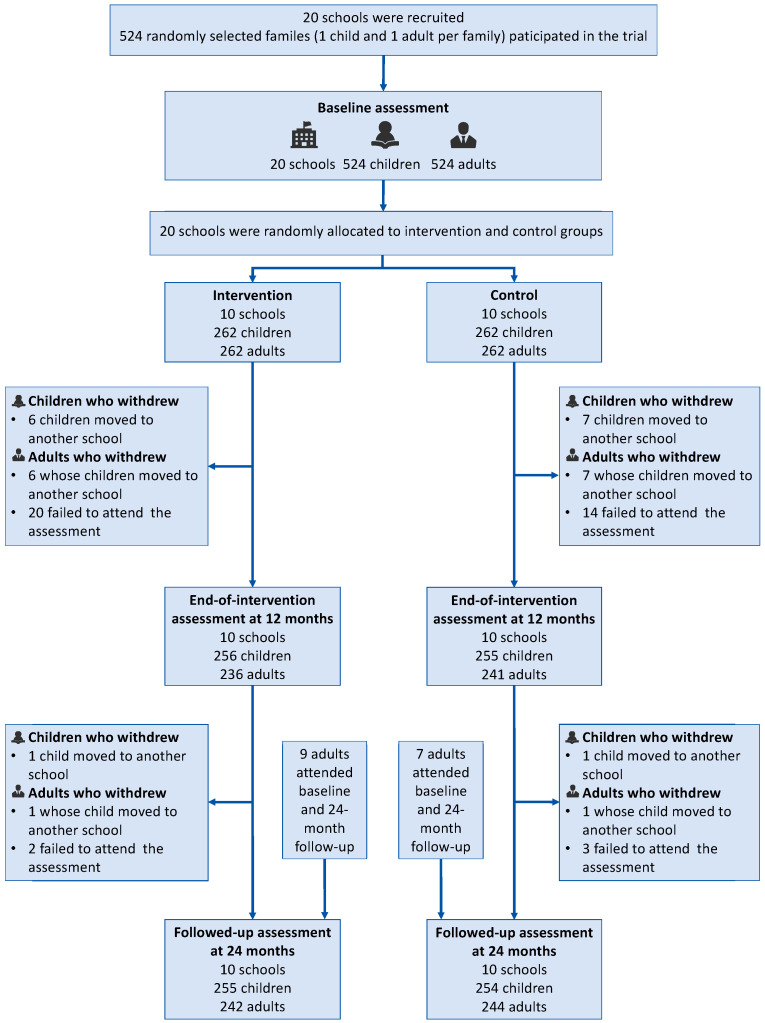
Flowchart of the follow-up sampling and analyses.

A total of 524 children and adults from 20 primary schools completed the baseline assessment. The mean baseline ages of children in the intervention and control group were 9.10 years (SD = 0.35) years and 9.22 years (SD = 0.34) years, respectively. For adults, the average age was 40.96 years (SD = 11.07) in the intervention group and 41.01 years (SD = 11.03) in the control group. At the 24-month follow-up, 509 children and 486 adults completed the assessment, accounting for 97.1% and 92.7% of the baseline participants. Follow-up rates for children in the intervention and control groups were 97.3% (255/262) and 96.9% (254/262) and for adults were 92.4% (242/262) and 93.1% (244/262), respectively (Figure 1). Participants who self-reported hypertension were confirmed to be taking antihypertensive medication at the time of blood pressure measurement. The baseline characteristics of the study participants are presented in Table 1.

**Figure 2:**
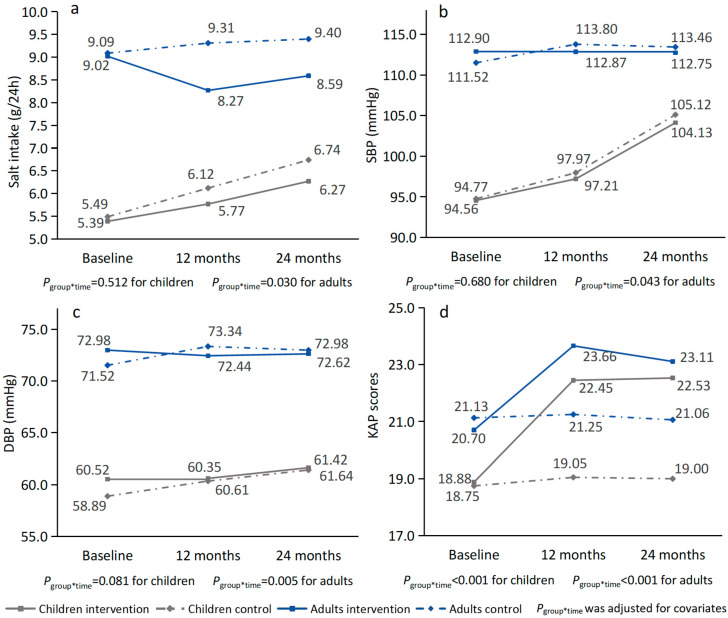
Unadjusted average salt intake (**a**), systolic blood pressure (SBP) (**b**), diastolic blood pressure (DBP) (**c**), and salt-related KAP scores (**d**) by group and time in children and adults.

As illustrated in the line graphs of the unadjusted average salt intake, SBP, DBP, and salt-related KAP by group and visit for both children and adults (Figure 2), significant differences in the two-year trajectories of these variables were observed between the intervention and control groups in adults (all *p <* 0.05), while among schoolchildren, only salt-related KAP scores differed significantly between groups (*p <* 0.001). The sample sizes used for analysis are provided in Table S4 and detailed values of these variables at baseline, 12 months, and 24 months are presented in Table S5.

As shown in Figure 2 and Table 2, at 24 months, the mean 24 h salt intake among adults was 8.59 (SD = 3.42) g/24 h in the intervention group and 9.40 (SD = 3.50) g/24 h in the control group; among schoolchildren, it was 6.27 (SD = 2.49) g/24 h and 6.74 (SD = 3.11) g/24 h, respectively. After adjusting for covariates, the 24 h salt intake among schoolchildren significantly increased from baseline to 24 months in both the intervention group (mean change: 0.56 g/24 h; 95% CI: 0.10 to 1.01, *p =* 0.017) and control group (mean change: 0.90 g/24 h; 95% CI: 0.44 to 1.36, *p <* 0.001). However, the adjusted mean difference in changes between the two groups at 24 months versus baseline was −0.34 g/24 h (95% CI: −0.94 to 0.26, *p =* 0.265) and was −0.09 g/24 h (95% CI: −0.69 to 0.51, *p =* 0.775) at 24 months versus 12 months. As detailed in Table 3, for adults, the adjusted mean difference in salt intake change between the intervention and control groups was −0.72 g/24 h (95% CI: −1.48 to 0.05, *p =* 0.065) at 24 months versus baseline and 0.29 g/24 h (95% CI: −0.50 to 1.08, *p =* 0.468) at 24 months versus 12 months, following an observed intervention effect of −1.01 g/24 h at the end of the intervention. In the intervention group, 12.75%, 17.76%, and 15.72% of adults met the WHO recommended salt intake (≤5 g/day) at baseline, 12 months, and 24 months, respectively, with no significant change over time (χ^2^ = 2.63, *p* = 0.269). In the control group, the corresponding proportions were 10.71%, 10.67%, and 9.79%, respectively, also showing no statistically significant variation (χ^2^ = 0.14, *p* = 0.933).

The SBP significantly increased from 12 to 24 months in both groups among schoolchildren, while among adults, it plateaued during the same period (Figure 2 and Table S5). Among schoolchildren, the mean SBP increased from baseline by 5.10 mmHg (95% CI: 3.06 to 7.14, *p <* 0.001) in the intervention group and by 5.67 mmHg (95% CI: 3.91 to 7.44, *p <* 0.001) in the control group at 24 months. In adults, following an adjusted reduction in SBP of 2.33 mmHg after the 12-month intervention, the mean difference of change in SBP after adjusting for covariates was −1.88 (95% CI: −3.83 to 0.08, *p =* 0.060) mmHg at 24 months versus baseline and 0.45 (95% CI: −1.54 to 2.43, *p =* 0.658) mmHg at 24 months versus 12 months. No significant difference was found in the change of salt intake and SBP in either children or adults at 24 months compared to baseline or 12 months after adjusting for confounding factors, nor were there significant differences in urinary potassium, sodium-to-potassium ratio, or DBP, except for adult DBP when comparing 24 months versus baseline.

An increase of KAP scores was observed at 12 months and 24 months in both children and adults, with children demonstrating better maintenance of these improvements at 24 months, whereas adults exhibited a slight decline in KAP scores from 12 to 24 months (Figure 2 and Table S5). As detailed in Table 2, having adjusted for covariates, the intervention group showed significantly elevated KAP scores compared to the control group both at 12 months and at 24 months versus baseline, although the difference changed from 3.20 (95% CI: 2.43 to 3.97, *p <* 0.001) to 3.35 (95% CI: 2.58 to 4.12, *p <* 0.001) in children, and the difference decreased from 2.87 (95% CI: 1.91 to 3.83, *p <* 0.001) to 2.50 (95% CI: 1.55 to 3.45, *p <* 0.001) in adults. Changes in scores of knowledge, attitudes, and behaviors showed similar trends to those observed in the overall KAP scores.

**Figure 3:**
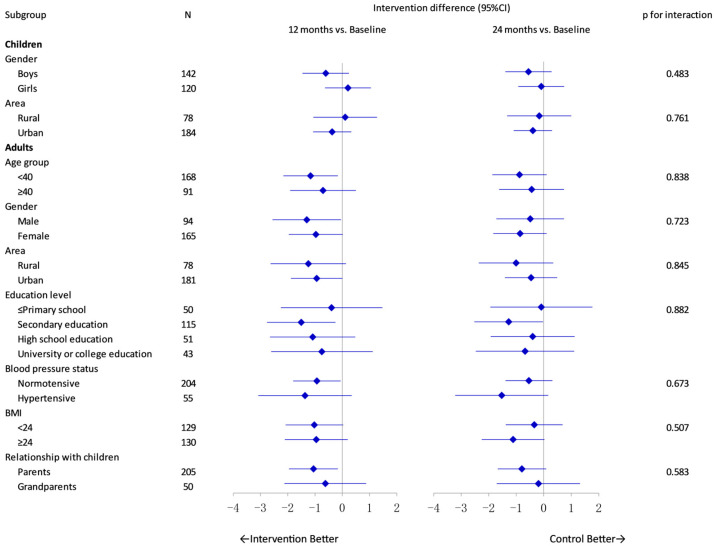
Adjusted intervention effect on 24 h salt intake by subgroup with 12/24-month follow-up versus baseline.

Sensitivity analyses of salt intake and other urinary measurements yielded similar results with those of the primary analyses (Tables S6 and S7). As shown in Figure 3, subgroup analyses revealed no significant heterogeneity of intervention effect on 24 h salt intake across different subgroups among both children and adults. Similar patterns were observed for its effects on SBP and DBP (Figures S3 and S4). Among adults with a secondary education, the two-year trend of salt intake differed significantly between the intervention and control groups (*p =* 0.039), suggesting a more favorable intervention effect in this subgroup (Figure S2). Additionally, the effect on SBP appeared more pronounced among adults who were overweight (BMI ≥ 24) (Figure S3). However, these findings should be interpreted with caution due to the relatively small sample size within subgroups, which may limit the robustness of the results.

## 4. Discussion

Currently, salt-reduction and hypertension-control initiatives in China primarily rely on the basic public health services. While these services reach a broad population, they face significant challenges in effectively engaging households. To address this gap, our research team developed EduSaltS, an innovative and comprehensive intervention based on previous studies. This study assessed the long-term effectiveness of EduSaltS through a one-year follow-up after a cluster randomized controlled trial conducted in real-world settings in Ganzhou, Jiangxi Province. Our findings showed that, among children, reductions in salt intake and blood pressure at the 24-month follow-up were not statistically significant. However, there was a trend suggesting continued improvement in salt reduction compared to the immediate post-intervention results. Among adults, the effects on salt reduction and blood pressure control diminished one year after the intervention. Despite this attenuation, improvements in knowledge, attitude, and practices (KAP) were sustained in both children and adults, although a slight decline in KAP scores was observed among adults at 24 months.

Schools are widely regarded as effective platforms for delivering health education to both students and their families. In this study, the EduSaltS intervention significantly improved schoolchildren’s knowledge, attitudes, and behaviors related to salt reduction, with these gains largely sustained one year after the intervention concluded. However, the intervention did not produce significant changes in children’s 24 h salt intake or blood pressure. This suggests that while enhancing knowledge is achievable, translating it into behavioral changes—particularly dietary sodium reduction—remains challenging, consistent with findings from previous studies [23,24]. Children aged 9–11 years have limited autonomy over their diets, as their eating habits are heavily influenced by their parents and the broader environment [25,26]. This may partly explain the lack of significant reduction in salt intake observed in this group [27,28]. Furthermore, this age group is undergoing a critical stage of growth and development, during which physiological needs may lead to naturally increased salt intake and higher blood pressure. Such factors, along with individual variability, may have attenuated the measurable effects of the intervention.

Although the reduction in children’s 24 h salt intake at the end of the intervention was not statistically significant, a slight but non-significant increase in effect was observed one year later (from −0.25 g/day at 12 months to −0.34 g/day at 24 months), suggesting a potential longer-term benefit. This finding is consistent with evidence indicating that improvements in knowledge, attitudes, and behaviors established during childhood may have enduring impacts on health into adolescence and adulthood [29].

Among adults, knowledge and attitudes related to salt reduction improved significantly following the EduSaltS intervention and remained elevated one year post intervention. However, the initial positive effects on the 24 h salt intake and blood pressure diminished at the 24-month follow-up. Adults in the intervention group were unable to maintain salt-reduction behaviors effectively over time. Specifically, the attenuation of the intervention effect was primarily driven by a rebound in salt intake within the intervention group, whose average daily intake increased from 8.27 g/24 h at 12 months to 8.59 g/24 h at 24 months, while the control group’s intake remained relatively unchanged. This pattern highlights a key challenge in long-term behavior change: sustaining health-promoting actions without continuous support. The difficulty adults face in maintaining lower salt consumption over time is well documented, with previous studies consistently identifying behavior maintenance as a major barrier in salt-reduction intervention [21,30]. During the EduSaltS intervention, a suite of integrated strategies—including online and offline health education, collaborative goal setting, active parent–school partnerships, and child-led encouragement—proved effective in mobilizing adults to adopt healthier dietary habits [17], but these support measures were not sustained after the intervention period. However, the relative stability of salt consumption in the control group over two years suggests minimal change in the broader food environment. As current food environments continue to promote high-salt diets [31], the lack of sustained motivation and support may hinder long-term adherence to salt-reduction behaviors and attenuate the lasting effects of the intervention.

Sustaining salt-reduction behaviors and achieving long-term impact require a coordinated approach that includes supportive environments, awareness-raising strategies, and effective monitoring systems. However, individuals living in high-sodium dietary environments face sustained barriers. The widespread presence of hidden salt in processed and packaged foods, along with growing inequalities within food systems, undermines personal efforts reduce sodium intake [32], limiting the lasting effects of dietary interventions. Longitudinal studies have highlighted the importance of intervention duration in maintaining the effectiveness of salt-reduction programs, showing that extended interventions can result in more sustained reductions in 24 h urinary sodium excretion as well as continued improvements in salt-related knowledge and attitudes [33]. In addition, emerging evidence increasingly supports the use of mHealth technologies as powerful tools to facilitate long-term behavior change. These platforms provide real-time self-monitoring, personalized feedback, and ongoing engagement, which can help individuals maintain healthier dietary habits over time [11,34].

It is important to note that this study coincided with major changes in China’s COVID-19 prevention policies, transitioning from strict home confinement in 2022 to return to normal life in 2023. These substantial lifestyle and psychological shifts may have influenced participants’ dietary behaviors. Although not directly measured in this study, such factors could have contributed to changes in salt intake and blood pressure. Furthermore, in our study, the persistence of behavioral knowledge without corresponding physiological improvement suggests a gap between knowledge and action. This disconnect may stem from limited motivation and self-efficacy, sociocultural norms surrounding communal meals, and environmental factors promoting high-salt diets. Informal interviews with families and teachers during the follow-up revealed that after the EduSaltS intervention ended, the app was no longer used, school-based salt-reduction activities were not maintained, and only a small number of intervention schools occasionally used electronic program materials for routine health education. The discontinuation of school-based mobilization and parent–school engagement, combined with the persistent high-salt dietary environments, likely impeded the translation of knowledge into sustained behavior change and weakened the long-term impact among adults.

This study also has several limitations. First, potential confounding factors affecting dietary intake and blood pressure, such as access to healthcare and lifestyle or psychological changes associated with shifts in COVID-19 policies, were not comprehensively assessed. Second, reliance on the self-reported KAP questionnaire responses may have introduced information bias. Third, the one-year follow-up period may be insufficient to fully capture the long-term effects on salt intake and blood pressure, particularly among growing children, and it is limited by the absence of continuous dynamic data. Nevertheless, adherence to standardized protocols across all three evaluation rounds and consistently high follow-up rates (above 90%) help mitigate these limitations and support the validity of the comparative analyses.

In summary, hypertension remains one of the leading global public health challenges, contributing to approximately 10.8 million preventable deaths each year [2]. The rapid advancement of digital health technologies presents new opportunities to strengthen behavioral interventions by integrating proven behavior-change models with innovative digital tools. While EduSaltS effectively promoted family-based salt reduction through the “small hands guiding big hands” strategy in real-world settings, additional efforts are needed to support the sustained behavior changes in adults and to achieve long-term reductions in salt intake and associated blood pressure outcomes.

## 5. Conclusions

The one-year follow-up after the EduSaltS cluster randomized controlled trial conducted in real-world settings demonstrated slightly attenuated effects on reducing salt intake and blood pressure among adults. Nevertheless, sustained improvements in salt-related knowledge, attitudes, and practices were observed in both children and adults. Additionally, the consistent reduction in salt intake among children, although modest, underscores the potential long-term benefits of the intervention and its cumulative effects over time. Future initiatives and public health efforts should focus on creating supportive environments and leveraging innovative digital health technologies to reinforce behavioral change and promote sustained salt reduction.

## Figures and Tables

**Table 1:** Basic characteristics at baseline.

Character	Control	Intervention	Total
**Cluster level**			
N of schools, by location, n (%)			
Zhanggong District	4 (40.00)	4 (40.00)	8 (40.00)
Nankang District	3 (30.00)	3 (30.00)	6 (30.00)
Xinfeng County	2 (20.00)	2 (20.00)	4 (20.00)
Yudu County	1 (10.00)	1 (10.00)	2 (10.00)
Meal provision			
Yes	6 (60.00)	6 (60.00)	12 (60.00)
No	4 (40.00)	4 (40.00)	8 (40.00)
N of families	262	262	524
Outdoor temperature (°C), mean (SD)	24.81 (2.21)	23.02 (1.68)	23.93 (2.16)
**Children level**			
Gender, n(%)			
Boys	146 (55.73)	142 (54.20)	288 (54.96)
Girls	116 (44.27)	120 (45.80)	236 (45.04)
Age mean (SD)	9.22 (0.34)	9.10 (0.35)	9.16 (0.35)
Weight mean (SD)	29.30 (6.70)	28.46 (5.95)	28.88 (6.34)
BMI mean (SD)	15.81 (2.79)	15.93 (2.34)	15.87 (2.57)
Physical activity, n (%)			
Yes	96 (36.64)	91 (34.73)	187 (35.69)
No	166 (63.36)	171 (65.27)	337 (64.31)
**Adult level**			
Gender, n (%)			
Male	97 (37.02)	97 (37.02)	194 (37.02)
Female	165 (62.98)	165 (62.98)	330 (62.98)
Age (years), mean (SD)	41.01 (11.03)	40.96 (11.07)	40.99 (11.04)
Weight (kg), mean (SD)	59.49 (10.02)	59.98 (10.48)	59.74 (10.24)
BMI (kg/m^2^), mean (SD)	23.23 (3.20)	23.76 (3.26)	23.50 (3.24)
Relationship with children, n (%)			
Parents	217 (82.82)	207 (79.01)	424 (80.92)
Grandparents	43 (16.41)	50 (19.08)	93 (17.75)
Other	2 (0.76)	5 (1.91)	7 (1.34)
Physical activity, n (%)			
Yes	88 (33.59)	98 (37.40)	186 (35.50)
No	174 (66.41)	164 (62.60)	338 (64.50)
Education			
≤Primary school	39 (14.89)	50 (19.08)	89 (16.98)
Secondary education	106 (40.46)	117 (44.66)	223 (42.56)
High school education	57 (21.76)	51 (19.47)	108 (20.61)
College or above	60 (22.90)	44 (16.79)	104 (19.85)
Smoking			
Yes	49 (18.70)	52 (19.85)	101 (19.27)
No	213 (81.30)	210 (80.15)	423 (80.73)
Alcohol drinking, n (%)			
Never	151 (57.63)	158 (60.31)	309(58.97)
Occasional	97 (37.02)	96 (36.64)	193 (36.83)
Regular	14 (5.34)	8 (3.05)	22 (4.20)
Self-reported hypertension, n (%)			
Yes	20 (7.63)	20 (7.63)	40 (6.62)
No	242 (92.37)	242 (92.37)	484 (92.37)

Refers to our previous evaluation of effectiveness of the trial [18].

**Table 2:** Changes in 24 h salt intake, blood pressure, 24 h urinary measurements, and salt-related KAP in children based on intention-to-treat analysis.

	Intervention Group		Control Group		Adjusted Difference in Change (Intervention vs. Control)	
	Adjusted Difference (95%CI) ^†^	*p*	Adjusted Difference (95%CI) ^†^	*p*	Difference (95%CI) ^‡^	*p*
Salt intake (g/24 h)						
12 months vs. baseline	0.21 (−0.23 to 0.64)	0.353	0.46 (0.02 to 0.89)	0.039	−0.25 (−0.86 to 0.35)	0.412
24 months vs. baseline	0.56 (0.10 to 1.01)	0.017	0.90 (0.44 to 1.36)	<0.001	−0.34 (−0.94 to 0.26)	0.265
24 months vs. 12 months	0.35 (0.78 to −0.08)	0.109	0.44 (0.88 to 0.00)	0.049	−0.09 (−0.69 to 0.51)	0.775
SBP (mmHg)						
12 months vs. baseline	0.43 (−1.00 to 1.86)	0.554	1.10 (−0.33 to 2.53)	0.132	−0.67 (−2.27 to 0.94)	0.414
24 months vs. baseline	5.10 (3.06 to 7.14)	<0.001	5.67 (3.91 to 7.44)	<0.001	−0.57 (−2.22 to 1.08)	0.497
24 months vs. 12 months	4.67 (6.07 to 3.27)	<0.001	4.57 (5.82 to 3.33)	<0.001	0.10 (−1.56 to 1.75)	0.907
DBP (mmHg)						
12 months vs. baseline	−0.97 (−2.19 to 0.24)	0.115	0.41 (−0.81 to 1.62)	0.512	−1.38 (−2.75 to −0.02)	0.048
24 months vs. baseline	−1.05 (−2.77 to 0.68)	0.233	0.29 (−1.20 to 1.78)	0.702	−1.34 (−2.74 to 0.06)	0.061
24 months vs. 12 months	−0.07 (1.12 to −1.26)	0.903	−0.12 (0.94 to −1.17)	0.831	0.04 (−1.37 to 1.45)	0.954
Urinary sodium (mmol/24 h)						
12 months vs. baseline	3.52 (−3.91 to 10.94)	0.353	7.84 (0.39 to 15.28)	0.039	−4.32 (−14.64 to 6.00)	0.412
24 months vs. baseline	9.55 (1.74 to 17.35)	0.017	15.37 (7.51 to 23.23)	<0.001	−5.82 (−16.06 to 4.41)	0.265
24 months vs. 12 months	6.03 (13.40 to −1.35)	0.109	7.53 (15.04 to 0.03)	0.049	−1.50 (−11.80 to 8.80)	0.775
Urinary potassium (mmol/24 h)						
12 months vs. baseline	1.95 (−0.10 to 4.00)	0.062	1.02 (−1.04 to 3.08)	0.330	0.93 (−1.93 to 3.79)	0.524
24 months vs. baseline	3.19 (1.05 to 5.33)	0.004	2.24 (0.08 to 4.39)	0.042	0.95 (−1.89 to 3.78)	0.511
24 months vs. 12 months	1.24 (3.28 to −0.80)	0.234	1.22 (3.29 to −0.86)	0.250	0.02 (−2.83 to 2.87)	0.988
Sodium-to-potassium ratio						
12 months vs. baseline	−0.01 (−0.39 to 0.36)	0.941	0.26 (−0.12 to 0.64)	0.176	−0.27 (−0.80 to 0.25)	0.301
24 months vs. baseline	0.09 (−0.31 to 0.49)	0.650	0.38 (−0.02 to 0.78)	0.063	−0.29 (−0.81 to 0.23)	0.273
24 months vs. 12 months	0.11 (0.48 to −0.27)	0.576	0.12 (0.50 to −0.26)	0.535	−0.01 (−0.53 to 0.51)	0.958
Score of knowledge						
12 months vs. baseline	1.82 (1.49 to 2.14)	<0.001	0.16 (−0.16 to 0.49)	0.320	1.65 (1.20 to 2.10)	<0.001
24 months vs. baseline	1.71 (1.36 to 2.06)	<0.001	0.02 (−0.33 to 0.36)	0.932	1.69 (1.24 to 2.14)	<0.001
24 months vs. 12 months	−0.11 (0.22 to −0.43)	0.525	−0.15 (0.18 to −0.48)	0.373	0.04 (−0.41 to 0.50)	0.850
Score of attitudes						
12 months vs. baseline	0.47 (0.24 to 0.69)	<0.001	0.01 (−0.21 to 0.24)	0.897	0.45 (0.14 to 0.76)	0.004
24 months vs. baseline	0.54 (0.31 to 0.78)	<0.001	−0.03 (−0.26 to 0.21)	0.815	0.57 (0.26 to 0.88)	<0.001
24 months vs. 12 months	0.08 (0.30 to −0.15)	0.504	−0.04 (0.18 to −0.27)	0.709	0.12 (−0.19 to 0.43)	0.452
Score of behaviors						
12 months vs. baseline	1.19 (0.90 to 1.49)	<0.001	0.09 (−0.20 to 0.39)	0.542	1.10 (0.70 to 1.51)	<0.001
24 months vs. baseline	1.27 (0.95 to 1.59)	<0.001	0.18 (−0.14 to 0.50)	0.263	1.09 (0.68 to 1.50)	<0.001
24 months vs. 12 months	0.07 (0.37 to −0.22)	0.622	0.09 (0.39 to −0.21)	0.555	−0.02 (−0.42 to 0.39)	0.942
KAP scores						
12 months vs. baseline	3.48 (2.92 to 4.03)	<0.001	0.27 (−0.28 to 0.83)	0.334	3.20 (2.43 to 3.97)	<0.001
24 months vs. baseline	3.52 (2.93 to 4.12)	<0.001	0.17 (−0.43 to 0.77)	0.571	3.35 (2.58 to 4.12)	<0.001
24 months vs. 12 months	0.05 (0.60 to −0.51)	0.872	−0.10 (0.46 to −0.66)	0.725	0.15 (−0.63 to 0.92)	0.710

Adjusted for age, gender, BMI (body weight in children instead), district/county, physical activity, and education level (the education level of the familial participant was used instead for children). In adults, additional adjustments were made for smoking, alcohol consumption, and relationship with the child. Blood pressure values were further adjusted for outdoor temperature. ^†^ Comparison of the means between baseline and 12-month and 24-month follow-ups. Positive values = increases from baseline to 12/24-month follow-up; negative values = reductions from baseline to 12/24-month follow-up. ^‡^ Comparison between intervention and control groups in the changes from baseline and 12-month and 24-month follow-ups. Positive values = the intervention group had a greater increase or less decrease from baseline to 12/24-month follow-up than the control group; negative values = the intervention group had a greater decrease or smaller increase from baseline to 12/24-month follow-up than the control group.

**Table 3:** Changes in 24 h salt intake, blood pressure, 24 h urinary measurements, and salt-related KAP in adults based on intention-to-treat analysis.

	Intervention Group		Control Group		Adjusted Difference in Change (Intervention vs. Control)	
	Adjusted Difference (95%CI) ^†^	*p*	Adjusted Difference (95%CI) ^†^	*p*	Difference (95%CI) ^‡^	*p*
Salt intake (g/24 h)						
12 months vs. baseline	−0.78 (−1.33 to −0.23)	0.006	0.23 (−0.31 to 0.77)	0.405	−1.01 (−1.78 to −0.23)	0.011
24 months vs. baseline	−0.42 (−0.96 to 0.12)	0.128	0.30 (−0.24 to 0.83)	0.278	−0.72 (−1.48 to 0.05)	0.065
24 months vs. 12 months	0.36 (0.92 to −0.20)	0.212	0.07 (0.62 to −0.48)	0.814	0.29 (−0.50 to 1.08)	0.468
SBP (mmHg)						
12 months vs. baseline	0.62 (−1.06 to 2.31)	0.467	2.95 (1.26 to 4.64)	0.001	−2.33 (−4.26 to −0.40)	0.018
24 months vs. baseline	0.46 (−1.88 to 2.80)	0.699	2.34 (0.29 to 4.38)	0.025	−1.88 (−3.83 to 0.08)	0.060
24 months vs. 12 months	−0.16 (1.50 to −1.83)	0.847	−0.61 (0.86 to −2.08)	0.415	0.45 (−1.54 to 2.43)	0.658
DBP (mmHg)						
12 months vs. baseline	−0.45 (−1.75 to 0.85)	0.497	1.93 (0.63 to 3.24)	0.004	−2.38 (−3.87 to −0.90)	0.002
24 months vs. baseline	−0.40 (−2.22 to 1.42)	0.667	1.29 (−0.30 to 2.88)	0.112	−1.69 (−3.19 to −0.18)	0.028
24 months vs. 12 months	0.05 (1.34 to −1.24)	0.937	−0.65 (0.49 to −1.78)	0.266	0.70 (−0.84 to 2.23)	0.372
Urinary sodium (mmol/24 h)						
12 months vs. baseline	−13.27 (−22.70 to −3.85)	0.006	3.93 (−5.34 to 13.20)	0.405	−17.2 (−30.42 to −3.99)	0.011
24 months vs. baseline	−7.16 (−16.40 to 2.07)	0.128	5.06 (−4.09 to 14.21)	0.278	−12.23 (−25.23 to 0.78)	0.065
24 months vs. 12 months	6.11 (15.71 to −3.50)	0.212	1.13 (10.55 to −8.29)	0.814	4.98 (−8.48 to 18.43)	0.468
Urinary potassium (mmol/24 h)						
12 months vs. baseline	1.23 (−1.05 to 3.50)	0.289	−0.94 (−3.17 to 1.30)	0.412	2.16 (−1.03 to 5.35)	0.183
24 months vs. baseline	1.75 (−0.48 to 3.98)	0.123	0.36 (−1.85 to 2.57)	0.752	1.40 (−1.74 to 4.54)	0.383
24 months vs. 12 months	0.52 (2.84 to −1.79)	0.657	1.29 (3.57 to −0.98)	0.265	−0.77 (−4.02 to 2.48)	0.643
Sodium-to-potassium ratio						
12 months vs. baseline	−0.41 (−0.80 to −0.02)	0.038	0.21 (−0.17 to 0.59)	0.285	−0.62 (−1.17 to −0.07)	0.026
24 months vs. baseline	−0.16 (−0.54 to 0.22)	0.408	0.15 (−0.23 to 0.52)	0.448	−0.31 (−0.84 to 0.23)	0.262
24 months vs. 12 months	0.25 (0.65 to −0.15)	0.215	−0.06 (0.33 to −0.45)	0.753	0.31 (−0.24 to 0.87)	0.269
Score of knowledge						
12 months vs. baseline	1.38 (1.04 to 1.73)	<0.001	0.33 (−0.01 to 0.67)	0.059	1.05 (0.57 to 1.54)	<0.001
24 months vs. baseline	1.29 (0.95 to 1.63)	<0.001	0.23 (−0.11 to 0.57)	0.182	1.06 (0.58 to 1.54)	<0.001
24 months vs. 12 months	−0.09 (0.26 to −0.44)	0.607	−0.10 (0.25 to −0.45)	0.580	0.01 (−0.49 to 0.50)	0.981
Score of attitudes						
12 months vs. baseline	0.99 (0.60 to 1.38)	<0.001	−0.04 (−0.43 to 0.35)	0.825	1.03 (0.48 to 1.58)	<0.001
24 months vs. baseline	0.82 (0.43 to 1.21)	<0.001	−0.07 (−0.46 to 0.32)	0.716	0.89 (0.34 to 1.44)	0.002
24 months vs. 12 months	−0.17 (0.23 to −0.57)	0.403	−0.03 (0.37 to −0.42)	0.889	−0.14 (−0.7 to 0.42)	0.620
Score of behaviors						
12 months vs. baseline	0.60 (0.37 to 0.83)	<0.001	−0.19 (−0.42 to 0.04)	0.107	0.79 (0.46 to 1.12)	<0.001
24 months vs. baseline	0.33 (0.10 to 0.57)	0.005	−0.23 (−0.46 to 0.00)	0.053	0.56 (0.24 to 0.89)	0.001
24 months vs. 12 months	−0.27 (−0.03 to −0.50)	0.028	−0.04 (0.20 to −0.27)	0.754	−0.23 (−0.56 to 0.10)	0.179
KAP scores						
12 months vs. baseline	2.98 (2.29 to 3.66)	<0.001	0.11 (−0.57 to 0.78)	0.756	2.87 (1.91 to 3.83)	<0.001
24 months vs. baseline	2.44 (1.77 to 3.12)	<0.001	−0.06 (−0.73 to 0.62)	0.866	2.50 (1.55 to 3.45)	<0.001
24 months vs. 12 months	−0.53 (0.16 to −1.23)	0.130	−0.17 (0.52 to −0.85)	0.636	−0.37 (−1.34 to 0.61)	0.458

Adjusted for age, gender, BMI (body weight in children instead), district/county, physical activity, and education level (the education level of the familial participant was used instead for children). In adults, additional adjustments were made for smoking, alcohol consumption, and relationship with the child. Blood pressure values were further adjusted for outdoor temperature. ^†^ Comparison of the means between baseline and 12-month and 24-month follow-ups. Positive values = increases from baseline to 12/24-month follow-up; negative values = reductions from baseline to 12/24-month follow-up. ^‡^ Comparison between intervention and control groups in the changes from baseline and 12-month and 24-month follow-ups. Positive values = the intervention group had a greater increase or less decrease from baseline to 12/24-month follow-up than the control group; negative values = the intervention group had a greater decrease or smaller increase from baseline to 12/24-month follow-up than the control group.

## Data Availability

The data used or analyzed in this study are available from the corresponding author on reasonable request.

## Supplementary Materials

The following supporting information can be downloaded at https://www.mdpi.com/article/10.3390/nu17111845/s1. Table S1: Distribution of intervention and control groups in 20 public primary schools; Table S2: Scoring criteria for salt-related knowledge, attitudes, and behaviors of children; Table S3: Scoring criteria for salt-related knowledge, attitudes, and behaviors of adults; Table S4: Sample size included in each type of analysis at different time points; Table S5: Salt intake, blood pressure, 24 h urinary measurements, and salt-related KAP by group and visit in children and adults; Table S6: Salt intake and other 24 h urinary measurements in children based on sensitivity analysis; Table S7: Salt intake and other 24 h urinary measurements in adults based on sensitivity analysis; Figure S1: The evolution of studies from School-EduSalt and AppSalt to EduSaltS; Figure S2: Unadjusted average salt intake (g/24 h) by visit and subgroup among adults and children; Figure S3: Adjusted intervention effect on SBP by subgroup with 12/24-month follow-up vs. baseline; Figure S4: Adjusted intervention effect on DBP by subgroup with 12/24-month follow-up vs. baseline.
