# Supplementary material for: Sustained Effects of a Scaled-Up mHealth and School-Based Intervention for Salt Reduction (EduSaltS) in Schoolchildren and Their Families: 1-Year Follow-Up of a Cluster Randomized Controlled Trial
Source: Nutrients. 2025 May 28;17(11):1845. doi: 10.3390/nu17111845 (PMC12158195; doi:10.3390/nu17111845)
Supplement: Supplementary file 1 [file nutrients-17-01845-s001.zip › Supplementary Figures (S1-S4).pdf]

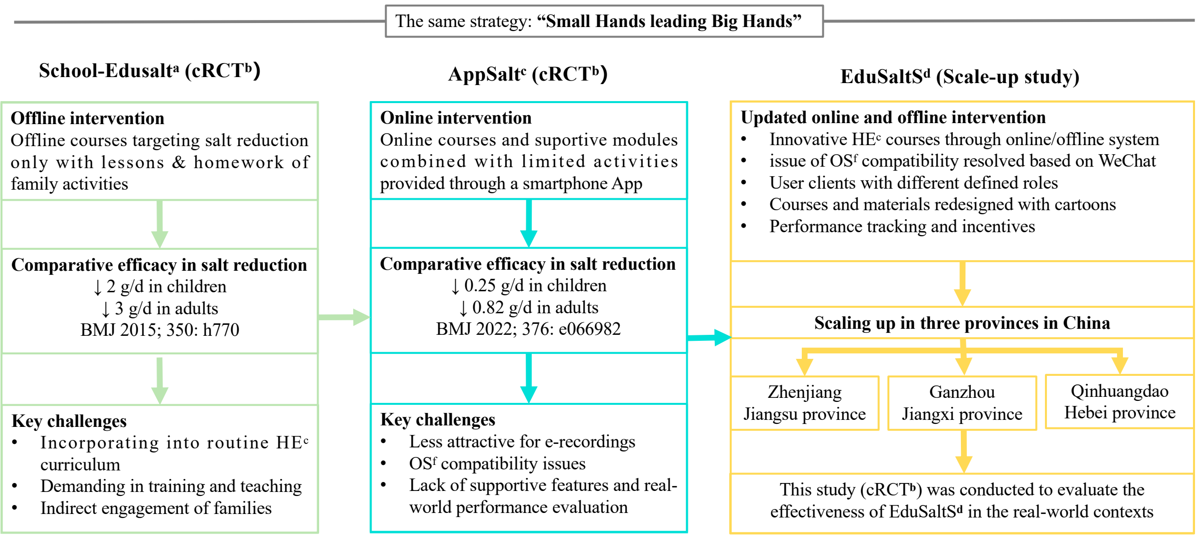

[a]School-EduSalt: A school-based education program to reduce salt intake in children and their families
[b]cRCT: Cluster randomized controlled trials
[c]AppSalt: An app based education programme to reduce salt intake
[d]EduSaltS: A scaled-up mHealth and school-based education program to reduce salt intake in China
[e]HE: Health education
[f]OS: Operation system

Figure S1. The evolution of studies from School-EduSalt, AppSalt to EduSaltS. (referred to the previous evaluation of effectiveness Paper [18])

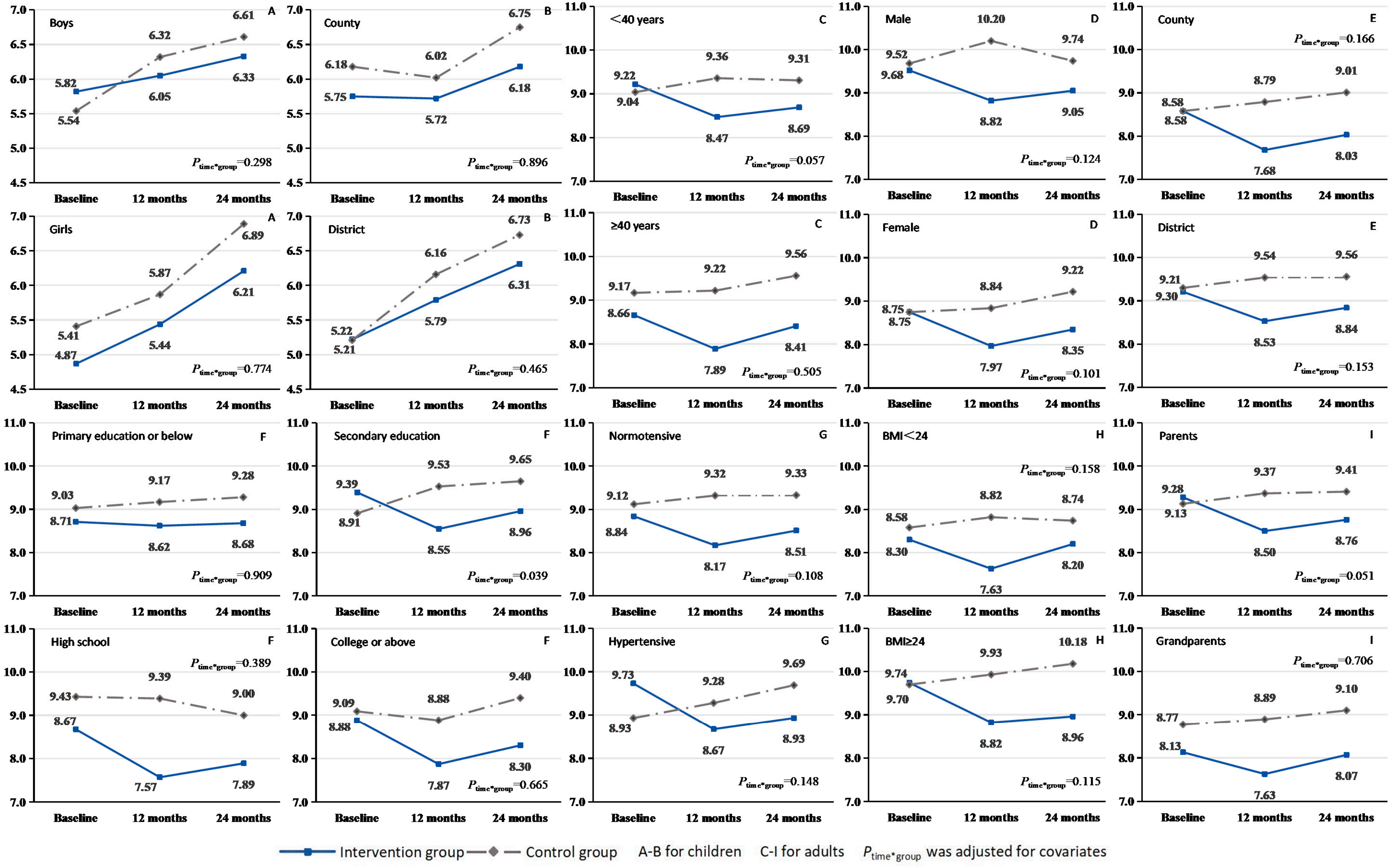

Figure S2. Unadjusted average salt intake (g/24h) by visit and subgroup among adults and children.

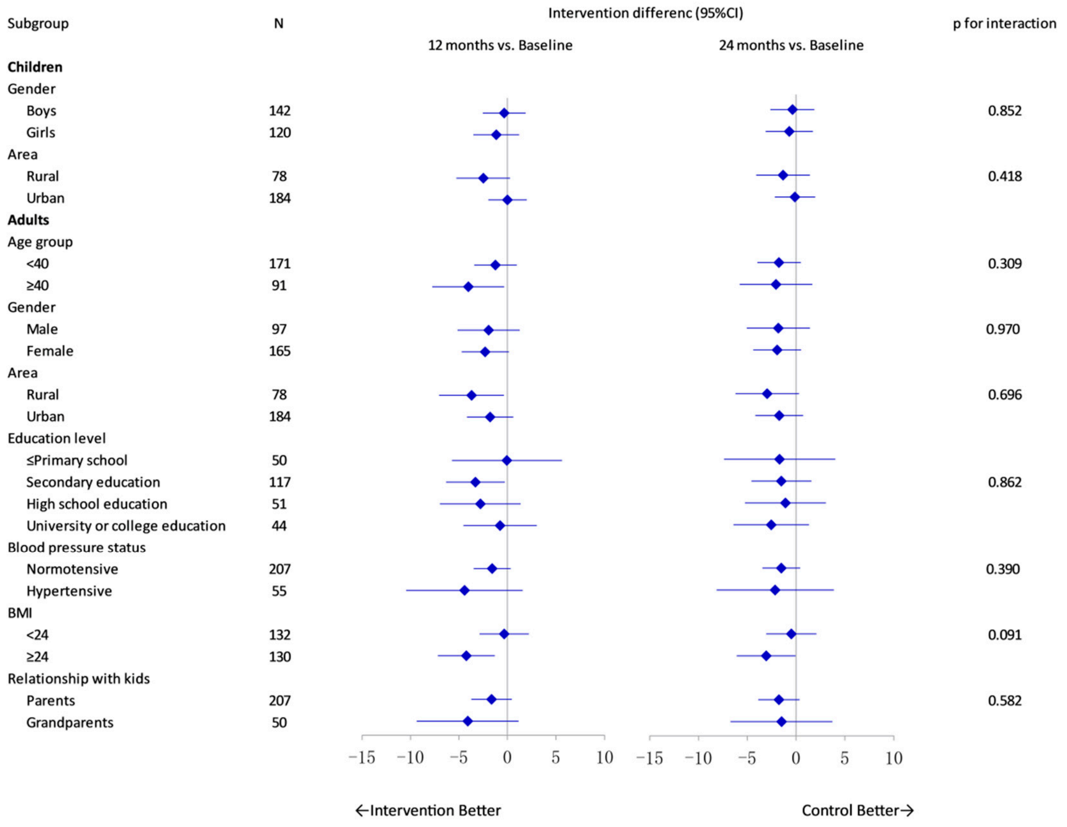

Figure S3. Adjusted intervention effect on SBP by subgroup with 12/24-month follow-up vs. baseline.

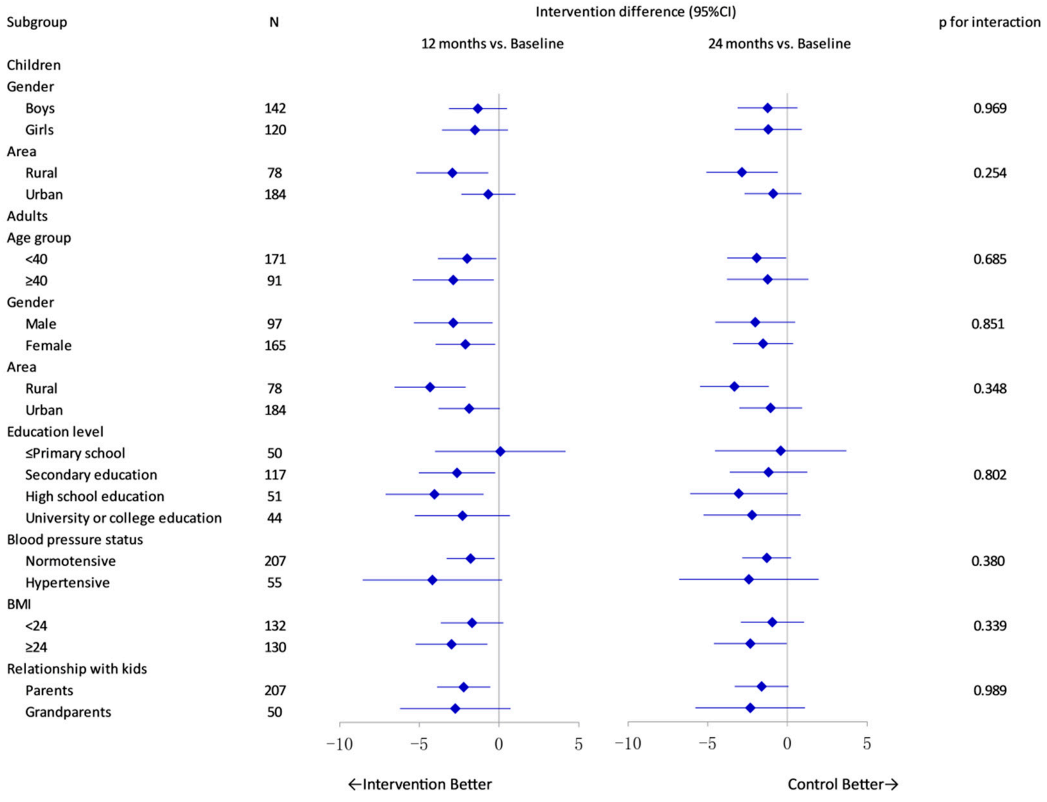

Figure S4. Adjusted intervention effect on DBP by subgroup with 12/24-month follow-up vs.

baseline.
