# Supplementary material for: Sustained Effects of a Scaled-Up mHealth and School-Based Intervention for Salt Reduction (EduSaltS) in Schoolchildren and Their Families: 1-Year Follow-Up of a Cluster Randomized Controlled Trial
Source: Nutrients. 2025 May 28;17(11):1845. doi: 10.3390/nu17111845 (PMC12158195; doi:10.3390/nu17111845)
Supplement: Supplementary file 1 [file nutrients-17-01845-s001.zip › Supplementary Tables (S1-S7).pdf]

Table S1. Distribution of intervention and control groups in 20 public primary schools.

District/County	Control Group	Intervention Group
Zhanggong District	Baiyun Primary School, Yangming Primary School, Railway Primary School, Second Primary School	Tianzhu Mountain Primary School, Shahe Central Primary School, Shashi Central Primary School, Zhangjiang Road Primary School
Nankang District	Dongshan Central Primary School, Ninth Primary School, Longling Central Primary School	Sixth Primary School, Fushi Central Primary School, Sanyi Central Primary School
Xinfeng County	Xinfeng Fifth Primary School, Xinfeng Ninth Primary School	Xinfeng Third Primary School, Hope Primary School
Yudu County	Changzhengyuan Red Army Primary School	Yudu Third Primary School

Table S2. Scoring criteria for salt related knowledge, attitudes and behaviors of children.

**Questions**	**scoring method**
**Knowledge**	
**K1.How many grams of salt are recommended for primary school students aged 7-11 to eat each day?**<br>1=≤4g<br>2=≤6g<br>3=≤10g<br>999=don't know	option 1 = 10 points; else = 0 points.

**K2.What problems can result from eating too much salt over a long period of time?**<br>1=Elevated blood pressure<br>2=Reduced blood pressure<br>3=No impact<br>999=Don't Know	option 1 = 10 points; else = 0 points.
**K3.Which item in the food nutrition label indicates the salt content?**<br>1=Energy<br>2=Protein<br>3=Fat<br>4=Carbohydrates<br>5=Sodium<br>999=Don't Know	option 5 = 10 points; else = 0 points.
**K4.Does eating less salt lead to the lack of strength?**<br>1=Yes<br>2=No<br>999=Don't Know	option 2 = 10 points; else = 0 points.
**K5.Does eating less salt cause hair to turn white?**<br>1=Yes<br>2=No<br>999=Don't Know	option 2 = 10 points; else = 0 points.
**K6.Which of the following foods or condiments have a higher salt content?(Please select all that apply)**<br>fine dried noodles/instant noodles/ham sausage/sauced beef/soy sauce/monosodium glutamate/spiced melon seeds/ preserved plum/soda biscuits/rice crust	Each option earns 1 point
**Attitudes**	
**A1.Do you think primary school students should eat more or less salt compared to adults?**	option 2 = 10 points; option 3 or 999 = 5 points;

1=More<br>2=Less<br>3=Same<br>999=Don't know	option 1 = 0 points.
**A2.What would you think if food were cooked using less salt at home?**<br>1=Less salt means it's not delicious<br>2=A little lighter is better<br>3=Not very concerned about saltiness	option 2 = 10 points; option 3 = 5 points; option 1 = 0 points.
**A3. What do you think of the taste of the food cooked at home?**<br>1=A bit salty<br>2=Moderate<br>3=Less salty	option 1 = 10 points; option 2 = 5 points; option 3 = 0 points.
**Behaviors**	
**B1. Is low-sodium salt currently used in your home?**<br>1=Using<br>2=Not using<br>3=Don't know what kind of salt is used at home<br>4=Never heard of low-sodium salt	option 3 = 10 points; option 2 = 5 points; option 1 = 0 points.<br>(option 2 or 3 indicates knowing what low-sodium salt is)
**B2.Have you reminded your parents of the following aspects?(Please select all that apply)**<br>1=Reducing salt and seasoning when cooking	Each option earns 2.5 point

2=Choosing snacks with relatively low salt content<br>3=Eating less pickled food<br>4=Asking preparing dishes with less salt when dining out	
**B3.How many times have you eaten out or ordered takeout over the past month?**<br>1=Almost every day<br>2=4 or more times per week<br>3=1-3 times per week<br>4=Almost none	option 4 = 10 points; option 3 = 6.6 points; option 2 = 3.3 points;option 1 = 0 points.
**B4.What do you think of the taste when dining out (including in restaurants, packaging, and takeout)?**[†]<br>1=More salty<br>2=Moderate<br>3=Less salty	option 3 = 10 points; option 2 = 5 points; option 1 = 0 points.
**B5.Do you proactively request less salt when dining out or ordering takeout?**[†]<br>1=Never request<br>2=Occasional request (1-2 times out of 10)<br>3=Sometimes request (3-5 times out of 10)<br>4=Frequently request (6-9 times out of 10)<br>5=Request each time	option 5 = 10 points; option 4 = 7.5 points; option 3 = 5 points; option 2 = 2.5 points; option 1 = 0 points.
**B6.In the past month, how did you eat salty snacks (including bread, biscuits, puffed food, preserved fruits, dried fruits baked with salt, dried meat, dried fish, dried tofu, cooked soy sauce meat, etc.)?**<br>1=Almost every day<br>2=3-5 days per week	option 4 = 10 points; option 3 = 6.6 points; option 2 = 3.3 points;option 1 = 0 points.<br>option 999 was calculated as option 4 (Mode).

3=1-2 days per week	
4=No more than once per week	
999=Don’t know	

†Those who have never eaten out or ordered takeouts in the past month were not included.

Score of knowledge = (K1+K2+K3+K4+K5+K6)/6. Score of attitudes = (A1+A2+A3)/3. Score of behaviors = (B1+B2+B3+B4+B5+B6)/(4+n); If the choice for B3 was 'Almost never' (both B5 and B6 have no answers), then n = 0; otherwise, n = 2. Total score = score of knowledge + score of attitudes+ score of behaviors.

Table S3. Scoring criteria for salt related knowledge, attitudes and behaviors of adults.

**Questions**	**scoring method**
**Knowledge**	
**K1.How many grams of salt should be consumed for healthy adults per day?**	option 1 = 10 points; else = 0 points.
1=≤5g	
2=≤8g	
3=≤10g	
999=don't know	
**K2.What problems can result from consuming too much salt over a long period of time?**	option 1 = 10 points; else = 0 points.
1=Elevated blood pressure	
2=Reduced blood pressure	
3=No impact	
999=Don't Know	
**K3.Which item in the food nutrition label indicates the salt content?**	option 5 = 10 points; else = 0 points.
1=Energy	
2=Protein	
3=Fat	
4=Carbohydrates	
5=Sodium	

999=Don't Know	
**K4.Does eating less salt leads to the lack of strength?**	option 2 = 10 points; else = 0 points.
1=Yes	
2=No	
999=Don't Know	
**K5.Does eating less salt cause hair to turn white?**	option 2 = 10 points; else = 0 points.
1=Yes	
2=No	
999=Don't Know	
**K6.Which of the following foods or condiments have a higher salt content?(Please select all that apply)**	Each option earns 1 point
fine dried noodles/instant noodles/ham sausage/sauced beef/soy sauce/monosodium glutamate/spiced melon seeds/ preserved plum/soda biscuits/rice crust	
**Attitudes**	
**A1.What would you think if food were cooked using less salt at home?**	option 2 = 10 points; option 3 = 5 points; option 1 = 0 points.
1=Less salt means it's not delicious	
2=Less salt is good for health	
3=Not very concerned about saltiness	
**A2.Are you willing to choose less salty diet?**	option 1 = 10 points; option 2 = 5 points; option 3 = 0 points.
1=Willing and believing that I can do it	
2=Willing, but not confident that I can do it	
3=Unwilling	
**Behaviors**	
**B1.What is your usual taste when eating?**	option 3 = 10 points; option 2 = 5 points; option 1 = 0

1=More Salty<br>2=Moderate<br>3=Less Salty	points.
**B2.How many times do you usually eat pickled food (such as salted vegetables, pickles, salted eggs, and sauced meat) in a week?**<br>1=Almost every day<br>2=3-5 days<br>3=1-2 days<br>4=No more than once<br>999=Don't Know	option 4 = 10 points; option 3 = 6.6 points; option 2 = 3.3 points;option 1 = 0 points.<br>option 999 was calculated as option 4 (Mode).
**B3.How often have you eaten out or ordered takeout over the past month?**<br>1=Almost every day<br>2=4 or more times per week<br>3=1-3 times per week<br>4=Almost none	option 4 = 10 points; option 3 = 6.6 points; option 2 = 3.3 points;option 1 = 0 points.
**B4.Do you proactively request less salt when dining out or ordering takeout?**[†]<br>1=Never<br>2=Occasionally (1-2 times out of 10)<br>3=Sometimes (3-5 times out of 10)<br>4=Frequently (6-9 times out of 10)<br>5=Every time	option 5 = 10 points; option 4 = 7.5 points; option 3 = 5 points; option 2 = 2.5 points; option 1 = 0 points.
**B5.Which of the following methods have you used to reduce salt when dining out or ordering**	Each choice earns 2.5 point

**takeout?† (Please select all that apply)**<br>1=Choose dishes that are steamed, boiled, braised and mixed<br>2=Try not to order salted, pickled, and marinated products<br>3=Rinse food with heavy oil and salt before eating<br>4=Not or try not to drink salty leftover soup	
**B6.Do you currently use low-sodium salt in your home?**<br>1=Yes<br>2=No<br>3=Don’t know<br>4=Haven’t heard of low-sodium salt	option 3 = 10 points; option 2 or 3 = 5 points; option 1 = 0 points.(option 2 or 3 indicates knowing what low-sodium salt is)

†Those who have never eaten out or ordered takeouts in the past month were not included.

Score of knowledge = (K1+K2+K3+K4+K5+K6)/6. Score of attitudes = (A1+A2+A3)/3. Score of behaviors = (B1+B2+B3+B4+B5+B6)/(4+n); If the choice for B3 was 'Almost never' (both B5 and B6 have no answers), then n = 0; otherwise, n = 2. Total score = score of knowledge + score of attitudes+ score of behaviors.

Table S4. Sample size included in each type of analysis at different time points.

		Intervention group			Control group		
		Baseline	12 months	24 months	Baseline	12 months	24 months
**Participants for each visit**							
	Children	262	256	255	262	255	254
	Adults	262	241	244	262	236	242
**24-h urine collection missing**							
	Children	0	0	0	0	0	0
	Adults	0	1	0	0	0	0
**Possibly incomplete 24-h urine**							
	Children	16	14	8	17	11	9
	Adults	11	26	15	10	11	7
**Primary analysis (Intention-to-treat analysis with complete 24 hour urine)**							
24-h urinary measurements	Children	246	242	247	245	236	243
	Adults	251	214	229	252	225	235
Blood pressure							
	Children	262	256	255	262	255	254
	Adults	262	241	244	262	236	242
**Including possibly incomplete 24 hour urine**							
24-h urinary measurements	Children	262	256	255	262	255	254
	Adults	262	240	244	262	236	242
Blood pressure							
	Children	262	256	255	262	255	254
	Adults	262	241	244	262	236	242

Table S5. Salt intake, blood pressure, 24-hour urinary measurements, and salt-related KAP by group and visit in children and adults.

	Intervention group						Control group					
	Baseline		12 months		24 months		Baseline		12 months		24 months	
	N	Mean (SD)	N	Mean (SD)	N	Mean (SD)	N	Mean (SD)	N	Mean (SD)	N	Mean (SD)
**Children**												
Salt intake (g/24h)	246	5.39(2.37)	242	5.77(2.53)	247	6.27(2.49)	245	5.49(2.85)	236	6.12(2.62)	243	6.74(3.11)
SBP (mm Hg)	262	94.56(9.37)	256	97.21(9.26)	255	104.13(9.62)	262	94.77(9.58)	255	97.97(9.76)	254	105.12(9.5)
DBP (mm Hg)	262	60.52(6.94)	256	60.61(6.1)	255	61.64(7.35)	262	58.89(8.02)	255	60.35(7.33)	254	61.42(6.71)
Urinary sodium (mmol/24h)	246	92.1(40.59)	242	98.66(43.18)	247	107.23(42.58)	245	93.78(48.68)	236	104.64(44.75)	243	115.14(53.14)
Urinary potassium (mmol/24h)	246	21.60(8.23)	242	23.95(11.07)	247	25.59(12.58)	245	23.98(11.43)	236	25.54(13.7)	243	27.29(15.49)
Sodium-to-potassium ratio	246	4.64(2.17)	242	4.71(2.37)	247	4.88(2.63)	245	4.45(2.49)	236	4.77(2.23)	243	4.93(2.72)
Score of knowledge	262	5.79(2.56)	256	7.68(2.27)	255	7.65(1.94)	262	5.64(1.89)	255	5.88(2.19)	254	5.81(2.01)
Score of attitudes	262	7.11(1.64)	256	7.57(1.23)	255	7.61(1.22)	262	7.05(1.59)	255	7.03(1.34)	254	6.97(1.41)
Score of behaviors	262	5.98(1.96)	256	7.20(2.07)	255	7.26(1.94)	262	6.06(1.94)	255	6.13(2.13)	254	6.23(2.12)
KAP score	262	18.88(4.37)	256	22.45(3.88)	255	22.53(3.66)	262	18.75(3.73)	255	19.05(3.8)	254	19.00(3.76)
**Adults**												
Salt intake (g/24h)	254	9.02(3.92)	214	8.27(3.47)	229	8.59(3.42)	252	9.09(3.73)	225	9.31(3.62)	235	9.40(3.50)
SBP (mm Hg)	262	112.90(16.60)	241	112.87(15.09)	244	112.75(14.51)	262	111.52(17.35)	236	113.8(17.38)	242	113.46(15.65)
DBP (mm Hg)	262	72.98(11.30)	241	72.44(10.18)	244	72.62(10.16)	262	71.52(10.80)	236	73.34(11.26)	242	72.98(10.62)
Urinary sodium (mmol/24h)	251	154.25(66.99)	214	141.37(59.36)	229	146.89(58.51)	252	155.32(63.71)	225	159.14(61.92)	235	160.64(59.78)
Urinary potassium (mmol/24h)	251	31.66(11.30)	214	32.95(13.51)	229	33.22(15.31)	252	33.87(14.91)	225	33.11(14.72)	235	34.36(14.02)
Sodium-to-potassium ratio	251	5.21(2.33)	214	4.79(2.39)	229	5.06(2.72)	252	5.15(2.60)	225	5.33(2.38)	235	5.30(2.77)
Score of knowledge	262	6.40(2.30)	241	7.78(2.18)	244	7.68(2.03)	262	6.36(2.18)	236	6.69(2.34)	242	6.59(2.32)
Score of attitudes	262	7.52(2.95)	241	8.49(2.42)	244	8.31(2.34)	262	7.79(2.65)	236	7.74(2.65)	242	7.70(2.59)

Score of behaviors	262	6.79(1.87)	241	7.40(1.64)	244	7.12(1.62)	262	6.98(1.84)	236	6.82(1.82)	242	6.77(1.67)
KAP score	262	20.70(5.23)	241	23.66(4.21)	244	23.11(4.17)	262	21.13(4.79)	236	21.25(5.00)	242	21.06(4.62)

Table S6. Salt intake and other 24-hour urinary measurements in children based on sensitivity analysis.

	**Intervention group**		**Control group**		**Adjusted difference in change (intervention vs. control）**	
	**Adjusted difference (95%CI)**[†]	***P***	**Adjusted difference (95%CI)**[†]	***P***	**Difference (95%CI)**[‡]	***P***
**Salt intake（g/24h）**						
12 months vs. baseline	0.12(-0.31 to 0.55)	0.575	0.41(-0.02 to 0.84)	0.062	-0.29(-0.88 to 0.31)	0.347
24 months vs. baseline	0.50(0.05 to 0.96)	0.031	0.88(0.43 to 1.34)	<0.001	-0.38(-0.98 to 0.22)	0.211
24 months vs. 12 months	0.38(0.81 to -0.05)	0.085	0.47(0.91 to 0.04)	0.033	-0.09(-0.69 to 0.50)	0.757
**Urinary sodium（mmol/24h）**						
12 months vs. baseline	2.11(-5.26 to 9.47)	0.575	6.99(-0.34 to 14.32)	0.062	-4.89(-15.08 to 5.30)	0.347
24 months vs. baseline	8.59(0.78 to 16.39)	0.031	15.09(7.27 to 22.91)	<0.001	-6.50(-16.69 to 3.68)	0.211
24 months vs. 12 months	6.48(13.86 to -0.90)	0.085	8.1(15.52 to 0.67)	0.033	-1.62(-11.85 to 8.62)	0.757
**Urinary Potassium（mmol/24h）**						
12 months vs. baseline	1.67(-0.35 to 3.68)	0.105	1.05(-0.96 to 3.05)	0.305	0.62(-2.17 to 3.41)	0.664
24 months vs. baseline	2.96(0.84 to 5.07)	0.006	2.48(0.36 to 4.60)	0.022	0.48(-2.32 to 3.27)	0.738
24 months vs. 12 months	1.29(3.31 to -0.73)	0.209	1.43(3.46 to -0.60)	0.166	-0.14(-2.95 to 2.66)	0.921
**Sodium-to-potassium ratio**						
12 months vs. baseline	-0.04(-0.41 to 0.33)	0.826	0.21(-0.15 to 0.58)	0.255	-0.25(-0.76 to 0.25)	0.327
24 months vs. baseline	0.09(-0.30 to 0.49)	0.642	0.31(-0.08 to 0.70)	0.121	-0.22(-0.73 to 0.29)	0.399
24 months vs. 12 months	0.13(0.50 to -0.23)	0.475	0.10(0.47 to -0.27)	0.603	0.04(-0.47 to 0.55)	0.890

Adjusted for age, gender, BMI (body weight in children instead), district or county, physical activity, and education level (the education level of the familial participant was used instead for children). In adults, additional adjustments were made for smoking, alcohol consumption, and relationship with the child. Blood pressure values were further adjusted for outdoor temperature.

†Comparison of the means between baseline, 12-month and 24-month follow-up. Positive values=increases from baseline to 12/24-month follow-up; negative values=reductions from baseline to 12/24-month follow-up.

‡Comparison between intervention and control groups in the changes from baseline, 12-month and 24-month follow-up. Positive values=the intervention group had a greater increase or less decrease from baseline to 12/24-month follow-up than the control group; negative values=the intervention group has a greater decrease or smaller increase from baseline to 12/24-month follow-up than the control group.

Table S7. Salt intake and other 24-hour urinary measurements in adults based on sensitivity analysis.

	**Intervention group**		**Control group**		**Adjusted difference in change (intervention vs. control）**	
	**Adjusted difference (95%CI)†**	***P***	**Adjusted difference (95%CI)†**	***P***	**Difference (95%CI)‡**	***P***
**Salt intake（g/24h）**						
12 months vs. baseline	-0.87(-1.4 to -0.33)	0.002	0.12(-0.41 to 0.65)	0.664	-0.99(-1.74 to -0.23)	0.010
24 months vs. baseline	-0.46(-0.99 to 0.07)	0.090	0.24(-0.29 to 0.76)	0.381	-0.69(-1.44 to 0.05)	0.069
24 months vs. 12 months	0.41(0.95 to -0.13)	0.138	0.12(0.66 to -0.42)	0.665	0.29(-0.47 to 1.06)	0.453
**Urinary sodium（mmol/24h）**						
12 months vs. baseline	-14.84(-23.98 to -5.70)	0.002	2.01(-7.06 to 11.08)	0.664	-16.85(-29.73 to -3.98)	0.010
24 months vs. baseline	-7.82(-16.87 to 1.23)	0.090	4.04(-4.99 to 13.07)	0.381	-11.86(-24.65 to 0.93)	0.069
24 months vs. 12 months	7.02(16.31 to -2.26)	0.138	2.03(11.22 to -7.17)	0.665	5.00(-8.07 to 18.06)	0.453
**Urinary Potassium（mmol/24h）**						
12 months vs. baseline	0.49(-1.70 to 2.69)	0.659	-1.44(-3.62 to 0.74)	0.194	1.93(-1.16 to 5.02)	0.220
24 months vs. baseline	1.48(-0.69 to 3.65)	0.182	0.35(-1.81 to 2.52)	0.750	1.13(-1.94 to 4.19)	0.472
24 months vs. 12 months	0.98(3.21 to -1.24)	0.386	1.79(4.00 to -0.42)	0.111	-0.81(-3.95 to 2.33)	0.613

**Sodium-to-potassium ratio**						
12 months vs. baseline	-0.41(-0.78 to -0.04)	0.031	0.22(-0.15 to 0.59)	0.239	-0.63(-1.15 to -0.11)	0.018
24 months vs. baseline	-0.20(-0.56 to 0.17)	0.283	0.03(-0.33 to 0.39)	0.872	-0.23(-0.75 to 0.29)	0.382
24 months vs. 12 months	0.21(0.58 to -0.17)	0.279	-0.19(0.18 to -0.56)	0.316	0.40(-0.13 to 0.92)	0.140

Adjusted for age, gender, BMI (body weight in children instead), district or county, physical activity, and education level (the education level of the familial participant was used instead for children). In adults, additional adjustments were made for smoking, alcohol consumption, and relationship with the child. Blood pressure values were further adjusted for outdoor temperature.

†Comparison of the means between baseline, 12-month and 24-month follow-up. Positive values=increases from baseline to 12/24-month follow-up; negative values=reductions from baseline to 12/24-month follow-up.

‡Comparison between intervention and control groups in the changes from baseline, 12-month and 24-month follow-up. Positive values=the intervention group had a greater increase or less decrease from baseline to 12/24-month follow-up than the control group; negative values=the intervention group has a greater decrease or smaller increase from baseline to 12/24-month follow-up than the control group.
